# IL-17 crosses the blood–brain barrier to trigger neuroinflammation: a novel mechanism in nitroglycerin-induced chronic migraine

**DOI:** 10.1186/s10194-021-01374-9

**Published:** 2022-01-03

**Authors:** Hao Chen, Xueqian Tang, Jin Li, Bangyan Hu, Wenqin Yang, Meng Zhan, Tengyun Ma, Shijun Xu

**Affiliations:** 1 School of Pharmacy, Chengdu University of Traditional Chinese Medicine, Chengdu, Sichuan 611137 People’s Republic of China; 2 Institute of Meterial Medica Integration and Transformation for Brain Disorders, Chengdu University of Traditional Chinese Medicine, Chengdu, Sichuan 611137 People’s Republic of China; 3 State Key Laboratory of Southwestern Chinese Medicine Resources, Chengdu, Sichuan 611137 People’s Republic of China

**Keywords:** Nitroglycerin, Migraine, IL-17, Blood–brain barrier, Neuroinflammation

## Abstract

**Background:**

Chronic migraine places a disabling burden on patients, which is extensively modeled by the nitroglycerin (NTG)-treated animal model. Although the NF-κB pathway is involved in an increase in CGRP levels and activation of the trigeminal system in the NTG model, the relationship between NTG and neuroinflammation remains unclear. This study aimed to optimize a chronic NTG rat model with hyperalgesia and the ethological capacity for estimating migraine therapies and to further explore the underlying mechanism of NTG-induced migraine.

**Methods:**

Rats were administered different doses of NTG s.c. daily or every 2 d; 30 min and 2 h later, the mechanical threshold was tested. After 9 d, the rats were injected with EB or Cy5.5 for the permeability assay. The other animals were sacrificed, and then, brainstem and caudal trigeminal ganglion were removed to test CGRP, c-Fos and NOS activity; Cytokines levels in the tissue and serum were measured by ELISA; and NF-κB pathway and blood–brain barrier (BBB)-related indicators were analyzed using western blotting. Immunohistochemistry was performed to observe microglial polarization and IL-17A^+^ T cell migration in the medulla oblongata.

**Results:**

NTG (10 mg/kg, s.c., every 2 d for a total of 5 injections) was the optimal condition, resulting in progressive hyperalgesia and migraine behavior. TNC neuroinflammation with increases in cytokines, CGRP and c-Fos and activation of the NF-κB pathway was observed, and these changes were alleviated by ibuprofen. Furthermore, NTG administration increased BBB permeability by altering the levels functional proteins (RAGE, LRP1, AQP4 and MFSD2A) and structural proteins (ZO-1, Occludin and VE-cadherin-2) to increase peripheral IL-17A permeation into the medulla oblongata, activating microglia and neuroinflammation, and eventually causing hyperalgesia and migraine attack.

**Conclusions:**

This study confirmed that NTG (10 mg/kg, s.c., every 2 d for a total of 5 injections) was the optimal condition to provoke migraine, resulting in mechanical hyperalgesia and observable migraine-like behavior. Furthermore, IL-17A crossed the blood–brain barrier into the medulla oblongata, triggering TNC activation through microglia-mediated neuroinflammation. This process was a novel mechanism in NTG-induced chronic migraine, suggesting that IL-17A might be a novel target in the treatment of migraine.

## Background

Chronic migraine is a disabling neurological condition affecting up to 2% of the global population, while limited therapeutic solutions are available. The clinical characteristics of migraine include frequent headaches, hyperalgesia to visual, auditory and olfactory stimuli, nausea and vomiting, which gravely compromise the quality of life of patients [4]. Although the mechanisms of chronic migraine remain entirely unclear, it has been demonstrated that inflammation and the activation and sensitization of the trigeminal system play crucial roles in the attack. Current studies have confirmed the presence of inflammation in patients with migraine, as several major cytokines, including TNF-α, IL-1β and IL-6, are altered in patients during migraine attacks and in attack-free intervals [13, 18]. Sterile meningeal neurogenic inflammation has been identified as an important step in patients with migraine characterized by visual aura attacks. The local inflammatory response is particularly initiated by calcitonin gene-related peptide (CGRP), pituitary adenylate cyclase-activating polypeptide (PACAP) and substance P (SP) from meningeal afferent fibers and subsequently causes vasodilatation with plasma protein extravasation and meningeal mast cell degranulation [9, 28, 29, 32]. Based on the current understanding of the premonitory systems, neurogenic neuroinflammation in the brainstem, hypothalamus and parenchyma, as another trigger and amplifier, has been accepted to be crucially involved in migraine [8, 19, 42]. Recent clinical observations have also provided supporting evidence for the presence of parenchymal neuroinflammation in patients with migraine [1]. Therefore, the pathophysiological mechanisms underlying migraine pain and related to these CNS components, particularly in neurogenic neuroinflammation, provide insights into potential new antimigraine targets.

Good animal models with the characteristics of migraine are indispensable tools to elucidate pathophysiological mechanisms and identify novel therapies for chronic migraine. Nitroglycerin (NTG) has a long history of use in the clinic as a treatment for unstable angina pectoris, myocardial infarction and heart failure. The interest of neurologists and headache specialists in NTG was stimulated by one of the most frequent side effects of nitrate therapy: headache. Intravenous NTG administration frequently induces a headache with moderate intensity, and is usually accompanied by throbbing, photophobia and phonophobia, as subjects with NTG-induced headache fulfill the International Headache Society (IHS) diagnostic criteria for migraine in a very high percentage of migraineurs [41]. Although headache indeed represents an annoying side effect of NTG treatment treating angina pectoris, this characteristic might be utilized to establish a migraine-like animal model since its response to NTG parallels headaches observed in human studies, such as photophobia, phonophobia, nausea and anxiety-like behaviors [14]. Moreover, the low cost and convenient establishment of the NTG model has led to its extensive utilization in migraine research. A mouse model with chronic intermittent administration of NTG was developed to further model the migraine-like characteristics of chronic migraine (CM); this model presents acute mechanical hyperalgesia with each exposure, as well as progressive and sustained basal hyperalgesia (1–15 mg/kg, i.p.) [12, 14]. These modeling conditions were subsequently extended to other species, although the conditions lacked optimization and conversion (approximately 10 mg/kg, i.p.) [30, 44]. Moreover, i.p. NTG injection causes severe visceral pain, which limits ethological observations. Therefore, the conditions of NTG administration must be optimized and screened in common experimental species, such as rats.

Nitric oxide (NO), a small gaseous signaling molecule, and its metabolites are widely accepted as important mediators of neurogenic inflammation and neuroinflammation involved in migraine, and these processes are mainly mediated by the NO-cGMP pathways, glutamatergic pathway and NF-κB pathway. Therefore, NTG, a NO-donating agent, has been recognized as a translational model that has been strongly implicated in the pathological mechanisms of migraine [22]. In human subjects, NTG-induced migraine is associated with an increase in the plasma CGRP concentration [27]; increased levels of CGRP, SP and PACAP were also reported in the plasma of rodents treated systemically with NTG [7, 26, 29]. Abundant reports have indicated that neurogenic inflammation caused by NTG-induced neural activation leads to migraine-like behavior and an increased sensitivity to pain in an NTG model. NO released from NTG initiate a rather slow process that likely involves the activation of the trigeminovascular system in the dura mater and the subsequent activation of trigeminal fibers and the trigeminal nucleus caudalis (TNC) [22]. Furthermore, the NTG-induced increase in NOS activity is implicated in higher basal CGRP levels and might activate the exudation of blood material synergistically, leading to neurogenic inflammation involving the NF-κB pathway [33, 54]. NTG-induced iNOS expression is also accompanied by an increase in NF-κB activity and levels of cytokines, such as TNF-α, IL-6 and IL-1β [18]. On the other hand, although neurogenic neuroinflammation is highly relevant to migraine, the underlying mechanisms by which NTG modulates this response are unclear. Neuroinflammation extensively involves blood–brain barrier (BBB) disruption in patients with multiple sclerosis, systemic lupus erythematosus and traumatic brain injury, in which CGRP and NO play pivotal roles [17, 38, 45, 49]. The medulla oblongata (MO) is one of the vital components in trigeminal nerve signal transduction from the TNC and contains a special BBB for sensing brain biochemical interactions, including NO, O_2_ and cytokines [52]. Although the MO has been reported to be activated during migraine attack, the trigger of MO activation remains unknown [43]. Some recent studies have suggested that microglial inflammatory polarization is involved in neuroinflammation of the MO [55] and NTG-induced migraine [36]. This evidence suggests that NTG-induced migraine is probably associated with alterations in blood–brain barrier (BBB) permeability induced by neuroinflammation and the vasodilatory effects of NO and CGRP. The mechanism of neuroinflammation must be investigated in NTG-administered model to reveal the possible mechanisms underlying the pathophysiology of migraine and to discover novel targets for identifying possible therapeutic strategies for migraine.

Therefore, this study aimed to optimize and screen NTG treatment conditions in order to establish a chronic migraine rat model with hyperalgesia and test the ethological capacity for assessing novel migraine-preventive therapies. Mechanistically, the effect of NTG on the BBB in the MO region was observed, and we subsequently explored the underlying mechanism of neuroinflammation induced by NTG-mediated BBB alteration.

## Methods

### Animal preparation

The adult male SPF-grade Sprague–Dawley rats (200 ± 20 g) used in the study were provided by the Experimental Animal Center at Byrness Weil Biotech Ltd. (Chongqing, China) and were maintained in plastic cages at 22 ± 2 °C on a 12 h light/dark cycle with free access to food and water in the Experimental Animal Center at Chengdu University of Traditional Chinese Medicine (Chengdu, China). The animal approval number provided by the Ethics Committee for Animal Experiments of the Institute of Materia Medica Integration and Transformation for Brain Disorders was IBD2020005. All studies were strictly performed in accordance with the international ethical guidelines and related ethical regulations of the IRB of the Institute of Materia Medica Integration and Transformation for Brain Disorders, Chengdu University of Traditional Chinese Medicine.

### Chemical reagents and antibodies

Nitroglycerin injection was purchased from Beijing Yimin Pharmaceutical Co., Ltd. (Beijing, China). DMSO, PBS and sodium dodecyl sulfate polyacrylamide gel kits were obtained from Solarbio (Beijing, China). Primary antibodies, including anti-c-Fos, anti-NF-κB p65, anti-NF-κB p-p65, anti-RAGE, anti-LRP1, anti-AQP4, anti-MFSD2A, anti-ZO-1, anti-Occludin, anti-VE-cadherin-2, anti-β-actin, and anti-Iba1, were furnished by Cell Signaling Technology (Beverly, MA, USA). anti-iNOS and anti-IL-17A antibodies were purchased from Abcam (Shanghai, China). Anti-fitc-CD_4_, Anti-alexa fluor 488-rabbit IgG, anti-cy3-mouse IgG, anti-mouse IgG and anti-rabbit IgG secondary antibodies were purchased from ABclonal (Wuhan, China).

### NTG administration

**Fig. 1:**
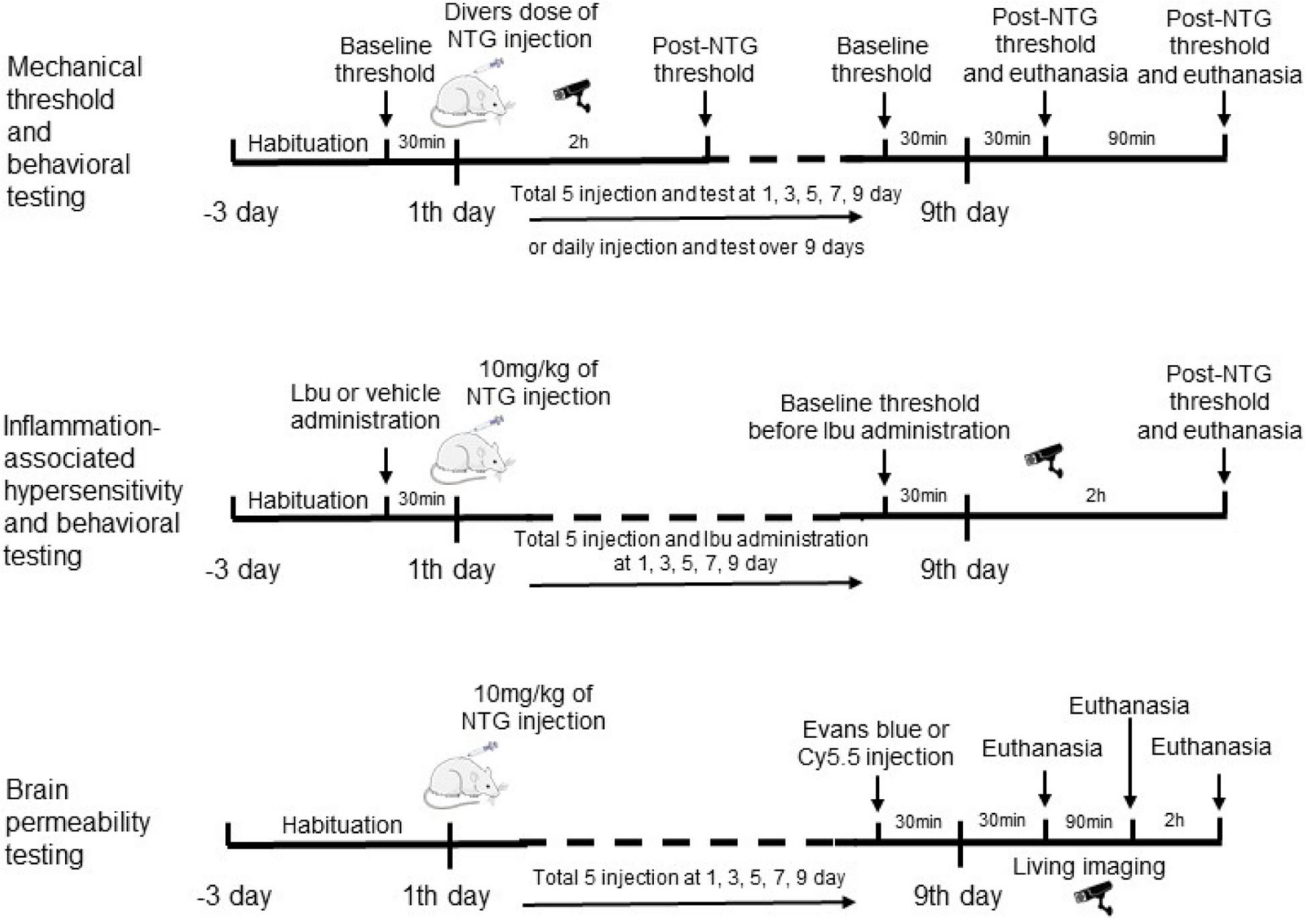
Time schedule of NTG administration in rats

NTG injection was prepared as a stock solution of 5.0 mg/mL. The vehicle control used in these experiments was 0.9% saline. NTG was freshly diluted in 0.9% saline to a series of predefined doses. All injections were administered at a 10 mL/kg volume. Unless indicated otherwise, animals were tested for baseline mechanical threshold responses immediately prior to the subcutaneous (s.c.) injection of NTG. The positive groups were orally administered ibuprofen 30 min before NTG injection. All groups were tested for the mechanical threshold 30 min or 2 h after NTG injection. For chronic experiments, testing occurred daily or every second day (5 test days total) for 9 d. Similarly, for the positive experiment, all rats were orally administered ibuprofen or vehicle every second day for 9 d (a detailed time schedule is shown in Fig. 1).

### Mechanical threshold test

The mechanical threshold for responses to punctate mechanical stimuli (mechanical hyperalgesia) was tested as described previously [21, 47]. Briefly, the plantar surface of the animal hindpaw was stimulated with electronic von Frey filaments (bending force ranging from 0 to 80 g), and the withdrawal threshold was measured by recording the instant withdrawal of the paw upon applying pressure from the tip. The average withdrawal reading of a minimum of three trials was recorded as the final value. Similarly, the tail surface was stimulated with an electronic analgesy-meter (bending force ranging from 0 to 600 g), and the response was defined as lifting or shaking of the tail or vocalization upon stimulation.

### Migraine-like behavioral test

The latency of ear redness, frequency of head scratching and number of cage-climbing events were measured in cubicles with a video camera (Epcbook, China) placed away from the cubicle in positions facing the subject. Briefly, before NTG injection, all rats were habituated to the cubicles for 30 min. Migraine-like behaviors were recorded over a 2-h NTG test period. Scratching and climbing behaviors and latency of ear redness were quantified based on the observations of the defined events (for details, see ML Chanda et al. [10], W Wen et al. [53]) in a blinded manner and counted by two colleagues from a digital video. The analysis of head scratching and cage climbing was valid only if the discrepancy between the two observers was less than 10% to avoid discrepancies between the two observers.

### Elisa

On the 9th day, half of the rats were sacrificed 30 min after NTG administration, and their serum, TNC and TG were collected. Then, the other rats were sacrificed 2 h after NTG administration, and serum and tissues were collected in the same manner. The CGRP content and NOS activity in tissues and the IL-17A, TNF-α, IL-6 and IL-1β contents in serum and tissues were determined using enzyme-linked immunosorbent assay (ELISA) kits (MultiSciences, China) according to the manufacturer’s instructions.

### qRT–PCR analysis

The mRNA samples were prepared and qRT–PCR was conducted as described previously [20]. Briefly, TNC and TG were collected in TRIzol (Invitrogen Life Technologies), and total RNA was extracted according to the manufacturer’s instructions. RNA samples were treated with RNase-free DNase I (Roche) to remove DNA contamination. The cDNA templates were produced from mRNA samples using the RevertAid First Strand cDNA Synthesis Kit (Thermo Scientific, USA). Quantitative determination of gene expression was performed using a Chromo 4 Detector (Bio-Rad, USA) with a two-step cycling protocol. Hypoxanthine-guanine phosphoribosyltransferase (HPRT) was used to normalize gene expression. qRT–PCR was conducted with cDNAs in duplicate 15 μL reactions using Maxima SYBR Green/ROX qPCR Master Mix (2X) (Thermo Scientific, USA). The reactions were incubated at 50 °C for 2 min and then at 95 °C for 10 min. A polymerase chain reaction cycling protocol consisting of 15 s at 95 °C and 1 min at 60 °C for 45 cycles was used for quantification. The relative expression levels were calculated according to the method described by Livak and Schmittgen34, and values were normalized to respective normal samples. The sequences of the primers used for qRT–PCR experiments were as follows: IL-17A, 5′-CTCAGACTACCTCAACCGTTCC-3′ and 5′-GTGCCTCCCAGATCACAGAAG-3′; α-CGRP, 5′-CCTGGTTGTCAGCATCTTGC-3′ and 5′-CACATTGGTGGGCACAAAG-3′; and NF-κB p65, 5′-GTACTTGCCAGACACAGACGA-3′ and 5′-CTCGGGAAGGCACAGCAATA-3′.

### Protein preparation and western blot analysis

Protein preparation and western blotting were conducted as described previously [53]. Briefly, the MO with TNC was collected and lysed in RIPA buffer containing phenylmethanesulfonyl fluoride and PhosSTOP (Solarbio, China). The supernatant containing proteins was collected and stored at − 80 °C until use. Protein concentrations were measured with a BCA kit (Beyotime, China). Equivalent amounts of the proteins were separated on SDS–PAGE gels and transferred to polyvinylidine difluoride membranes (Bio-Rad, USA). The membranes were blocked with 5% skim milk in Tris-buffered saline containing Tween 20 (0.5%) for 2 h. Subsequently, the membranes were incubated with the primary antibody at 4 °C overnight and incubated with the secondary antibody at 37 °C for 2 h. The HRP ECL system (Beyotime, China) was used to visualize the protein bands, and the gray values were then analyzed.

### Immunohistochemistry (IHC) analysis

Immunohistochemical staining was conducted as described previously [11]. Briefly, after embedding tissues in paraffin, the MO with TNC was cut into sections (5 μm), incubated with the designated primary antibodies, and visualized by incubating sections with the corresponding secondary antibodies before capturing images at a magnification of 200×/400× by confocal microscopy (Olympus, Japan).

### Live imaging

Live imaging was conducted as described previously [48]. Briefly, 200 μL of nonpenetrating fluorescent dye-loaded Cy5.5 (US Everbright, China) solution was injected into the tail vein of rats 30 min before NTG administration to determine the permeability of the brain. Fluorescence images of the rat brain were captured 4 h after NTG administration using an in vivo imaging system (Clairvivo OPT, SHIMADZU Corporation, Japan). Rats were fixed in dorsal positions to acquire optical images of the head.

### Evans blue assay

The Evans blue assay was conducted as described previously [2]. Briefly, a 4% Evans blue staining solution (Sigma–Aldrich) was freshly prepared and intravenously injected (4 mL/kg) into the tail vein of rats 30 min before NTG administration. Animals were euthanized 30 min and 2 h after the Evans blue injection. They were deeply anaesthetized and transcardially perfused with 4% paraformaldehyde. Images of the brain were captured using a digital camera (Olympus SZX16, Japan). TNC sections were frozen in OCT and photographed using a confocal microscope (Olympus, Japan).

### Statistical analysis

All data are presented as the means ± SD from three independent experiments. All data were determined to have a normal distribution using the Shapiro–Wilk test and statistically significant differences were analyzed using one-way analysis of variance (ANOVA) for multiple comparisons, and *p* values < 0.05 were considered significant.

## Results

### Optimization of conditions for NTG-induced migraine in rats

#### Mechanical threshold of rats with migraine induced by the administration of different doses of NTG for different periods

**Fig. 2:**
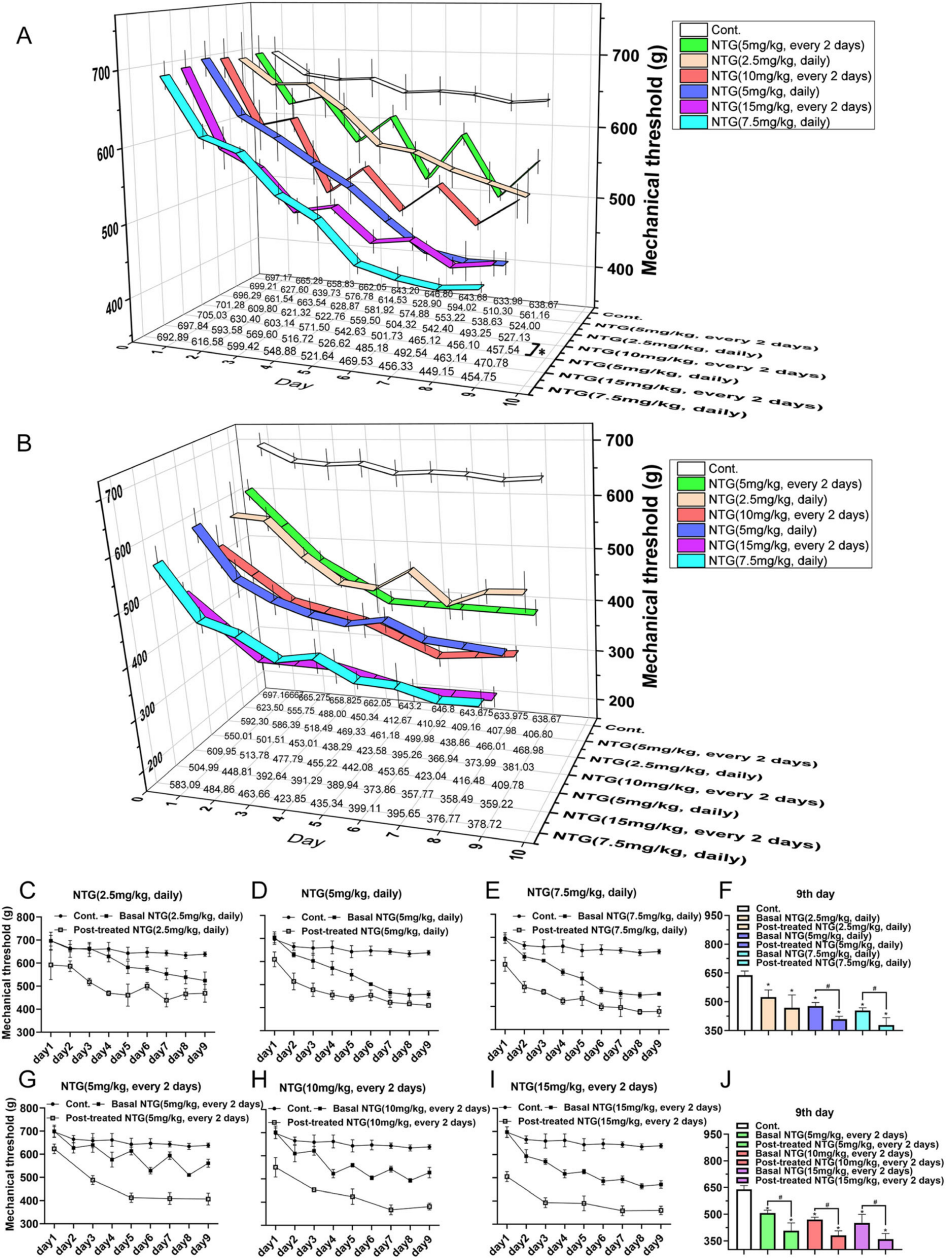
Analysis of the mechanical threshold of rats with NTG-induced migraine*.* Multiple NTG administrations evoked and sustained mechanical hyperalgesia. Rats were treated every 2 d with varying doses of NTG (0, 2.5, 5 and 7.5 mg/kg, s.c., daily or 0, 5, 10 and 15 mg/kg, s.c., every 2 d) for 9 d. (A) Basal mechanical threshold of all groups during NTG administration. (B) Posttreatment NTG mechanical threshold of all groups during NTG administration. (C-E) Mechanical threshold of the rats with migraine administered NTG s.c. daily for 9 d. (F) Mechanical threshold of the rats administered consecutive NTG s.c injections on the 9th day. (G-I) Mechanical threshold of the rats with migraine administered NTG s.c. every 2 d for 9 d. (J) Mechanical threshold of the rats s.c. administered intermittent NTG on the 9th day. Each dose group was significantly different from the vehicle group (*p* < 0.05), and each dose also produced significantly different results from the other doses (*p* < 0.05). NTG produces dose-dependent and persistent hyperalgesia in rats. Statistical analysis was performed using one–way ANOVA, *n* = 6, *p < 0.05 compared to the basal mechanical threshold

Based on the Meeh-Rubner formula and the literature [15, 40], the NTG equivalent dose for rats is 7 mg/kg, i.p., every 2 d. Unexpectedly, in the preliminary test, NTG-administered rats showed persistently crouched, spraining bodies and insensitive behavior after the first intraperitoneal injection of NTG, which impeded the ethological observation of the mechanical pain threshold. Therefore, subcutaneous injection was alternatively applied to deliver NTG because of its relatively mild irritation recorded in some migraine studies. We screened and optimized the modeling conditions by administering different dose doses (0–15 mg/kg) of NTG daily or every 2 d for 9 d, resulting in a total of 9 or 5 NTG injections/test days. Repetitive intermittent NTG administration over 9 days produced significant time- and dose-dependent chronic basal mechanical hyperalgesia, as assessed by testing prior to each administration of NTG (Fig. 2A). In addition, NTG evoked significant acute mechanical hyperalgesia in a dose-dependent manner on each test day (Fig. 2B). The greatest decreases in basal and posttreatment responses were observed in animals injected with 10 mg/kg NTG every 2 d (Fig. 2C-J). Thus, 10 mg/kg NTG was utilized in subsequent experiments.

#### Migraine-like behaviors of rats with migraine after administration of different doses of NTG for different periods

**Fig. 3:**
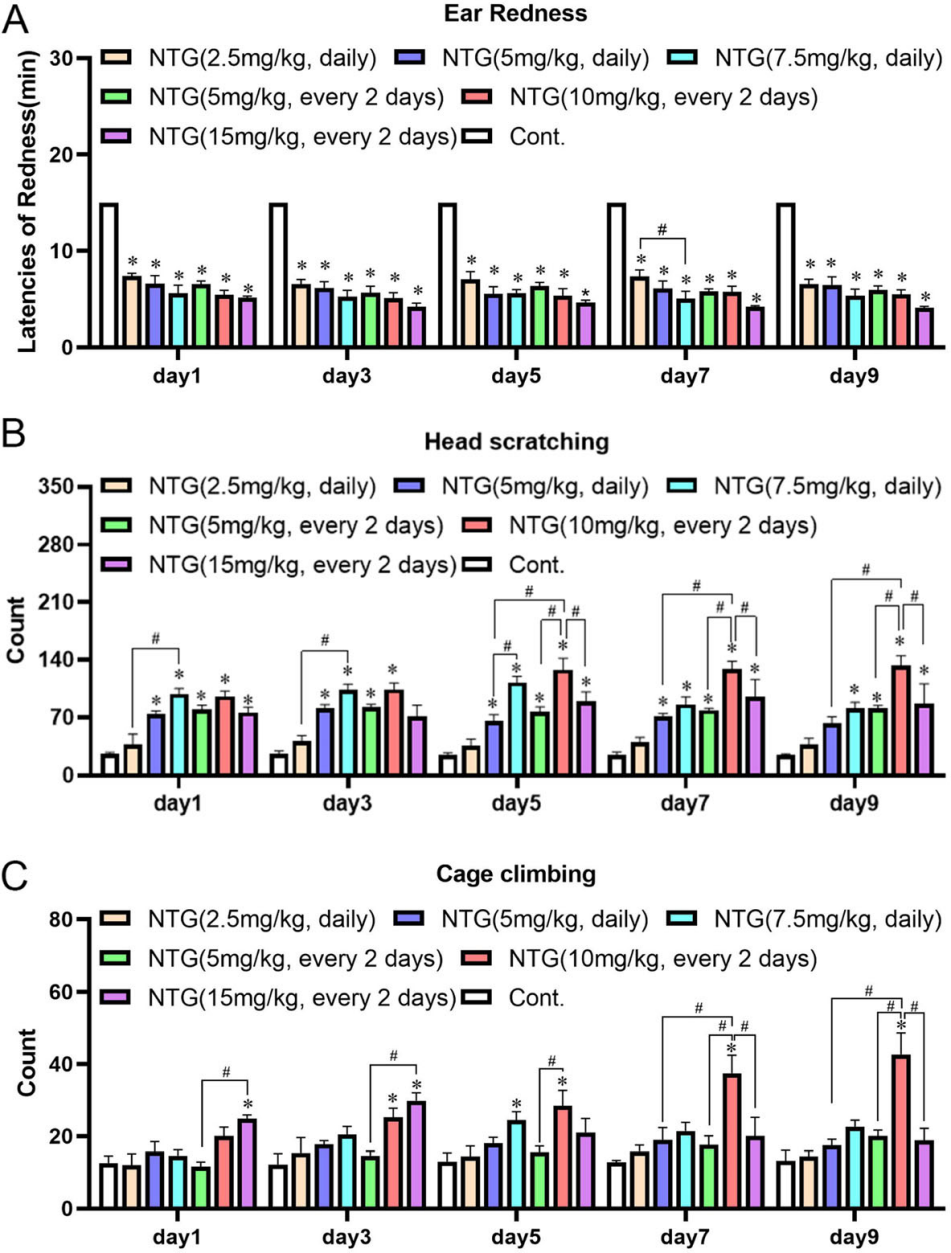
Migraine-like behaviors of rats with NTG-induced migraine*.* (A) Latency of ear redness in rats with NTG-induced migraine. (B) Frequency of head scratching in rats with NTG-induced migraine. (C) Number of cage climbing instances recorded for rats with NTG-induced migraine. The statistical analysis was performed using one–way ANOVA, n = 6, *p < 0.05 compared to the control group, and ^#^*p* < 0.05 compared to the indicated group

During migraine experiences, migraine-like behaviors of rats, such as head scratching and climbing, were exacerbated as a result of nociception, which has been extensively used to assess rodent migraine models. The results of behavioral tests indicated that the latency of ear redness was significantly shortened, while no difference was observed among the majority of groups (Fig. 3A). The number of head scratches was obviously increased within 120 min after NTG administration in the majority of groups. Similarly, the number of cage-climbing instances was also increased, especially after the administration of 10 mg/kg NTG every 2 d. The NTG administration group showed a prominent increase in this behavior, while the highest dose group exhibited insensitivity to cage climbing, similar to rats s.c. with NTG (Fig. 3B and C).

#### Migrainous mediator of migraine in rats treated with different doses of NTG for different times

**Fig. 4:**
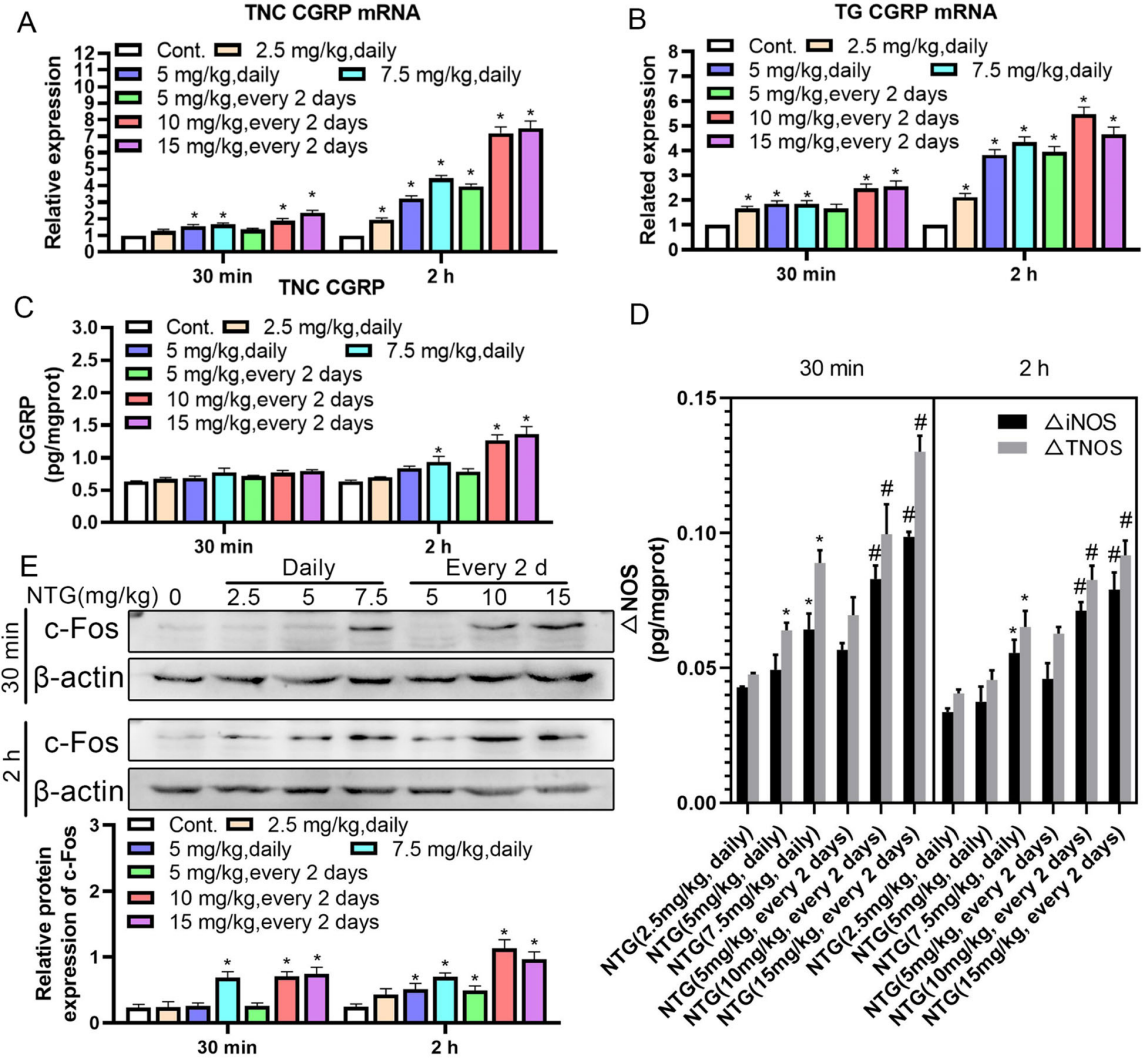
Migrainous mediators of the TNC and TG in rats with NTG-induced migraine. (A) The expression of the CGRP mRNA in the TNC of rats with NTG-induced migraine. (B) The expression of the CGRP mRNA in the TG of rats with NTG-induced migraine. (C) CGRP contents in the TNC of rats with NTG-induced migraine. *p < 0.05 compared to control group. (D) The variation in NOS levels in the TNC of rats with NTG-induced migraine. *p < 0.05 compared to the 2.5 mg/kg daily group; ^#^*p* < 0.05 compared to the 5 mg/kg every 2 d group. (E) The protein expression of the c-Fos in the TNC of rats with NTG-induced migraine. Statistical analysis was performed using one–way ANOVA, *n* = 3

Since CGRP, c-Fos and NOS are potent indicators of NTG-induced migraine, qRT–PCR was employed to measure the levels of the CGRP transcript in the TNC and TG. ELISA was used to monitor CGRP and NOS levels. Western blotting was employed to assess c-Fos expression on the 9th day. The qRT–PCR results indicated that various NTG administration protocols increased CGRP transcript levels in the TNC and TG (Fig. 4A and B). As predicted, the ELISA results revealed that NTG administration increased NOS levels in the TNC, while only 7.5 mg/kg daily and 10 and 15 mg/kg every 2 d groups showed significant increases in CGRP levels, especially in the 10 mg/kg every 2 d group at 2 h (Fig. 4C and D). Western blot results revealed that NTG induced c-Fos activation in the TNC (Fig. 4E). Altogether, 10 mg/kg NTG administered every 2 d is the most optimized modeling condition for chronic migraine in rats with mechanical hyperalgesia and ethological capacity.

### NTG-induced migraine is triggered by Neuroinflammation in the TNC

#### NTG induced neuroinflammation in the TNC and promoted microglial inflammatory polarization

**Fig. 5:**
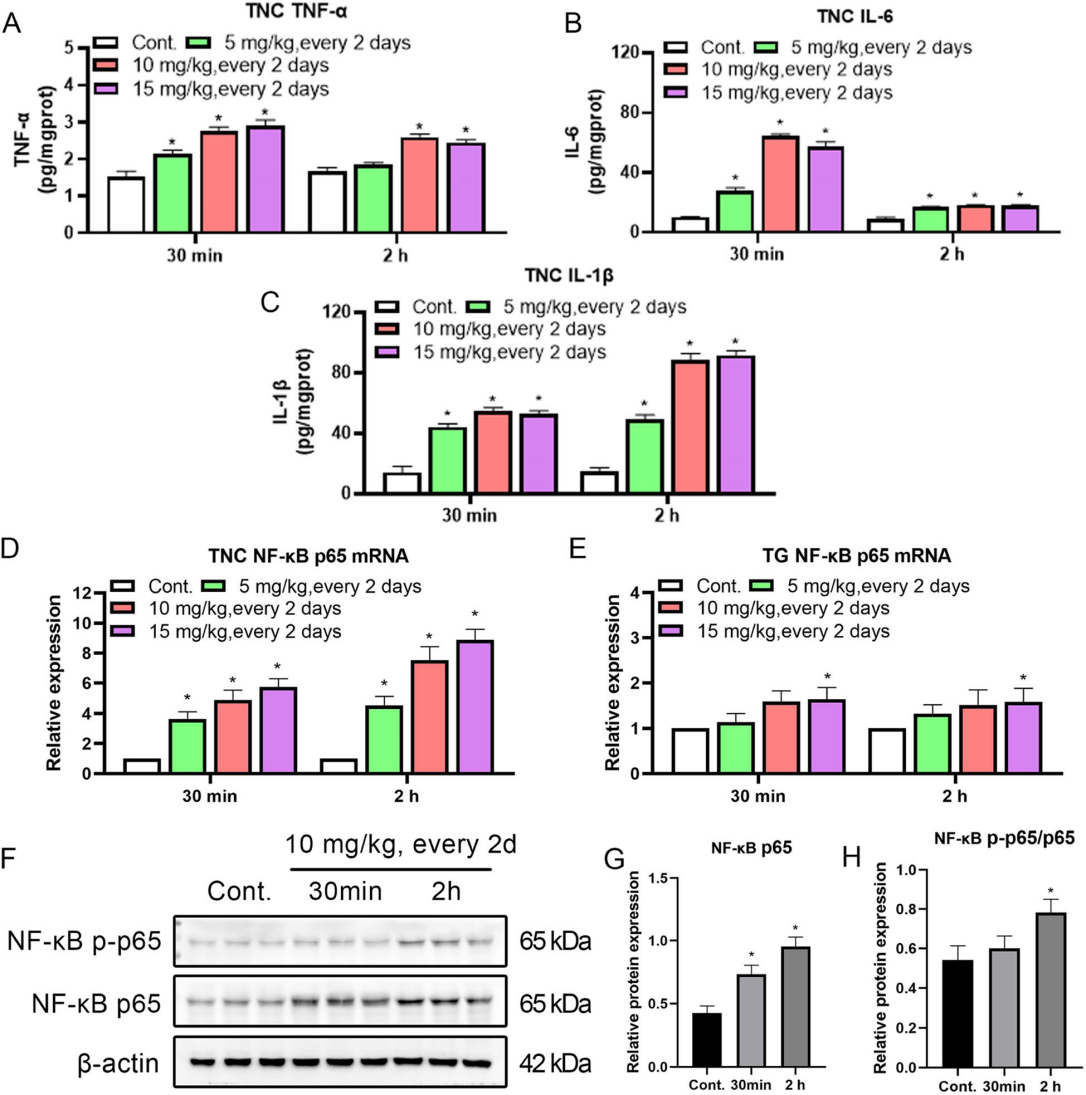
NTG increased the levels of inflammatory cytokines and activated the NF-κB signaling pathway in the TNC. (A-C) Content of TNF-α, IL-6 and IL-1β in the TNC of rats with NTG-induced migraine. (D and E) The expression of the NF-кB p65 mRNA in the TNC and TG of rats with NTG-induced migraine. (F-H) The levels of NF-κB p65, NF-κB p-p65 and the ratio of NF-κB p-p65/NF-κB p65 in the TNC of rats with NTG-induced migraine. Statistical analysis was performed using one–way ANOVA, n = 3, *p < 0.05 compared to the control group

**Fig. 6:**
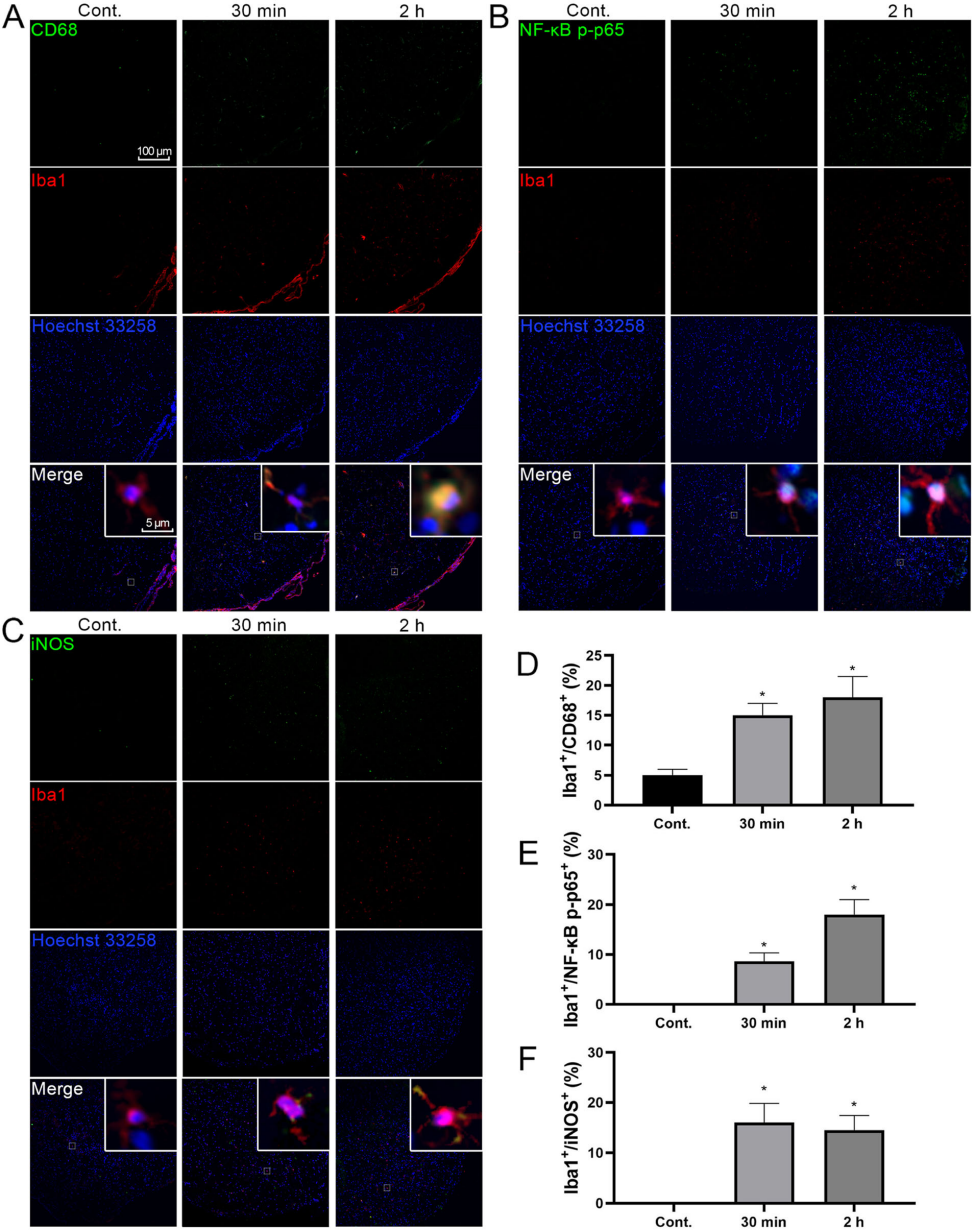
NTG promoted microglial inflammatory polarization in the TNC. (A) Images of Iba1 and CD68 costaining in the TNC of rats with NTG-induced migraine. (D) The ratio of Iba^+^/CD68^+^ cells. (B) Images of Iba1 and NF-κB p-p65 costaining in the TNC of rats with NTG-induced migraine. (E) The ratio of Iba^+^/ NF-κB p65^+^ cells. (C) Images of Iba1 and iNOS costaining in the TNC of rats with NTG-induced migraine. (F) The ratio of Iba^+^/ iNOS^+^ cells. Statistical analysis was performed using one–way ANOVA, n = 3, *p < 0.05 compared to the control group

Recent evidences suggest that neuroinflammation plays a pivotal role in migrainous hyperalgesia [18, 29]. The levels of the cytokines TNF-α, IL-6 and IL-1β, and NF-κB p65, an inflammation-related activated marker, were detected at 30 min and 2 h after NTG injection to investigate whether the TNC showed neuroinflammation hallmarks in the NTG administration model. The ELISA results showed that NTG increased the levels of inflammatory cytokines. The TNF-α and IL-6 levels were rapidly increased at 30 min, and the IL-1β level was subsequently increased at 2 h (Fig. 5A-C). The qRT–PCR results showed substantially increased levels of the NF-κB p65 transcript in the TNC at 30 min that persisted for 2 h compared to levels of the NF-κB p65 transcript in the TG (Fig. 5D and E). Western blot data further revealed that NTG increased NF-κB p65 expression and phosphorylation in the TNC, and this activation occurred sooner than the increase in CGRP levels (Fig. 4C; Fig. 5F and G), suggesting that NTG-induced hyperalgesia may be associated with inflammation in the TNC. TNC immunohistochemical staining also revealed that microglia, which function in immune surveillance and responses, were enriched in the TNC and its iNOS and NF-κB p65 were activated, suggesting that neuroinflammation was activated by microglia (Fig. 6).

#### Inhibiting neuroinflammation in the TNC alleviated NTG-induced hyperalgesia and migraine-like behaviors

**Fig. 7:**
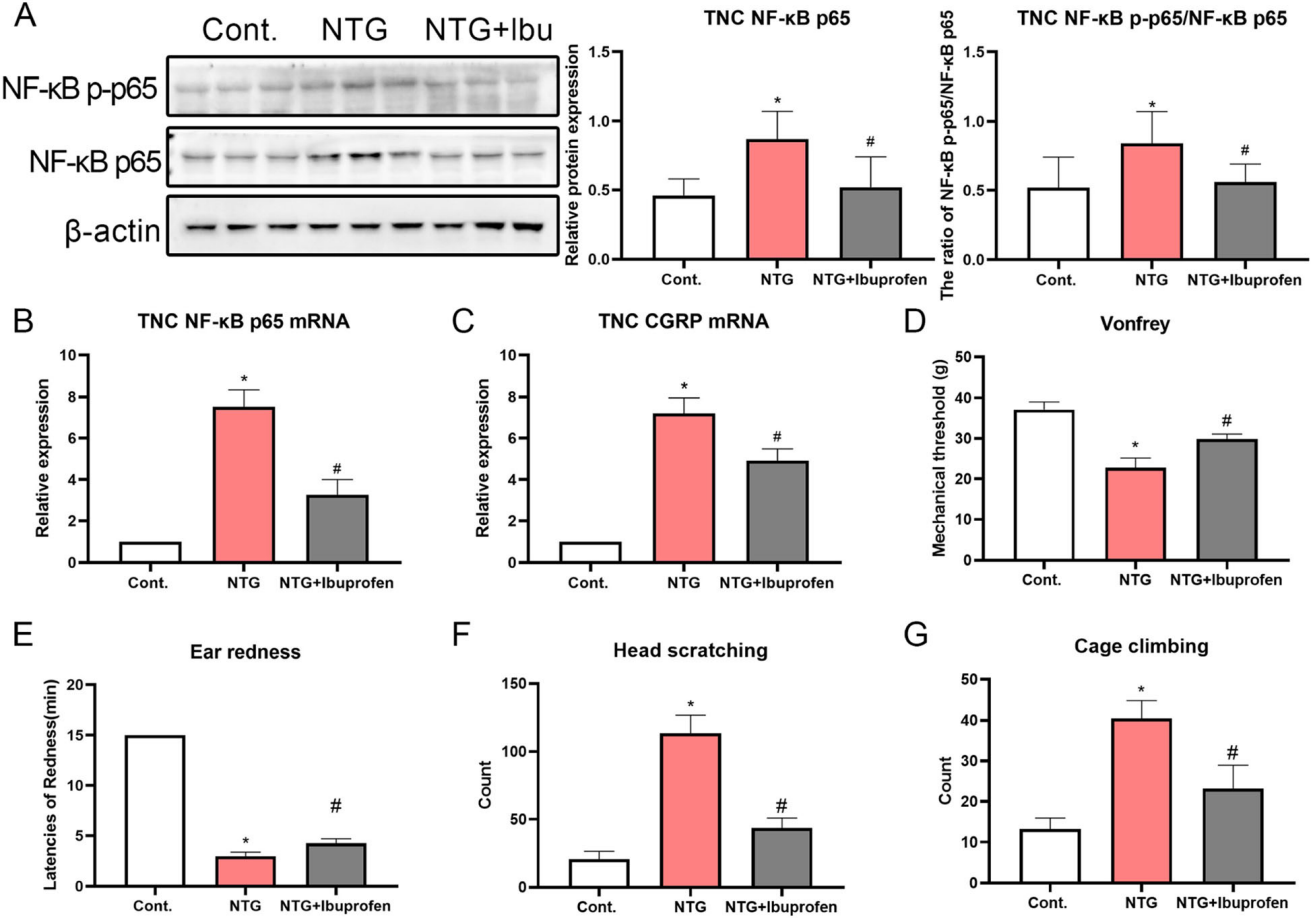
Inhibiting TNC neuroinflammation alleviated hyperalgesia and migraine-like behaviors in NTG-administered rats. (A) The expression of NF-κB p65 and the ratio of NF-κB p-p65/NF-κB p65 in the TNC after ibuprofen treatment. (B and C) The expression of the NF-кB p65 and CGRP mRNAs after lbu treatment. (D) Mechanical threshold recorded in NTG-treated rats after ibuprofen treatment. (E-G) Latency of ear redness, frequency of head scratching and number of cage climbing instances after ibuprofen treatment. Statistical analysis was performed using one-way ANOVA, n = 6, *p < 0.05 compared to the control group; ^#^*p* < 0.05 compared to the NTG group

Clinically, ibuprofen is extensively applied to relieve migraine symptoms due to its anti-inflammatory activity. Thus, ibuprofen was employed to treat NTG-induced inflammation and migraine. As expected, ibuprofen treatment reversed the increase in CGRP levels and activation of NF-κB signaling in the TNC (Fig. 7A-C). Ethological observations further confirmed that ibuprofen alleviated NTG-induced mechanical hyperalgesia (Fig. 7D) and migraine-like behavior (Fig. 7E, F and G). Based on these results, migraine symptoms in the NTG-administered model were induced by neuroinflammation in the TNC. The results that ibuprofen blocked NTG-induced inflammation were also supported by several previously reported findings, suggesting that the NTG-induced rat model could be utilized to evaluate migraine drugs with antiphlogistic activity.

Latency of ear redness in rats with NTG-induced migraine. (B) Frequency of head scratching in rats with NTG-induced migraine.

### IL-17A crosses BBB to trigger neuroinflammation in the TNC of rats with NTG-induced migraine

#### NTG administration increased BBB permeability

**Fig. 8:**
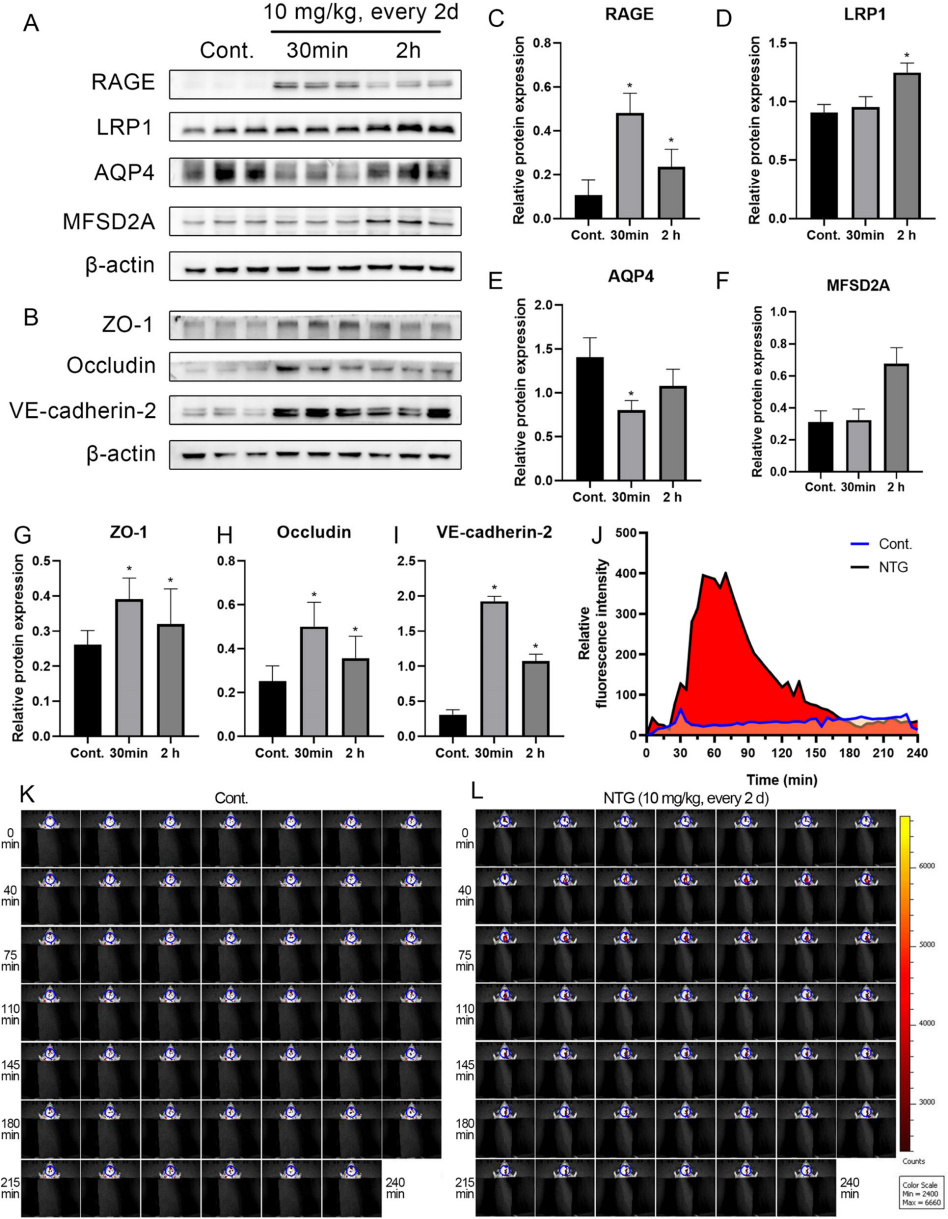
NTG administration increased BBB permeability. (A) NTG enhanced the transportation function of BBB. (C-F) The expression of RAGE, LRP1, AQP4 and MFSD2A in the TNC of rats with NTG-induced migraine. (B) NTG altered the structure of BBB. (G-I) The expression of ZO-1, Occludin and VE-cadherin-2 in the TNC of rats with NTG-induced migraine. (J-L) NTG increased the permeability of peripheral dye. After Cy5.5 circulation for 30 min, NTG was subcutaneously injected. A live imaging system recorded the fluorescence signals at 695 nm for 4 h at 5 min intervals. Statistical analysis was performed using one-way ANOVA, n = 3, *p < 0.05 compared to the control group

In the analysis of BBB-related proteins, the expression of RAGE/LRP1, a lipid transport functional protein, was inchoately increased after NTG injection. In contrast, the expression of AQP4/MFSD2A, an aqueous transport functional protein, was decreased (Fig. 8A, C-F). The structural proteins ZO-1, Occludin and VE-cadherin-2 were maintained at high levels, probably due to compensation for persistent NTG injection (Fig. 8B, G-I). Live imaging was employed to record the fluorescence intensity of an impervious far-infrared probe (Cy5.5) in the rat brain at 4 h and dynamically observe changes in BBB permeability after NTG injection. The fluorescence intensity was remarkably increased from 30 min to 180 min, and the peak fluorescence intensity was sustained from 45 min to 75 min after NTG injection, suggesting that the fast and transitory permeability generated by NTG might induce peripheral immune factor transport to the TNC (Fig. 8J-L).

#### NTG-induced BBB alterations increased peripheral IL-17A access to the TNC

**Fig. 9:**
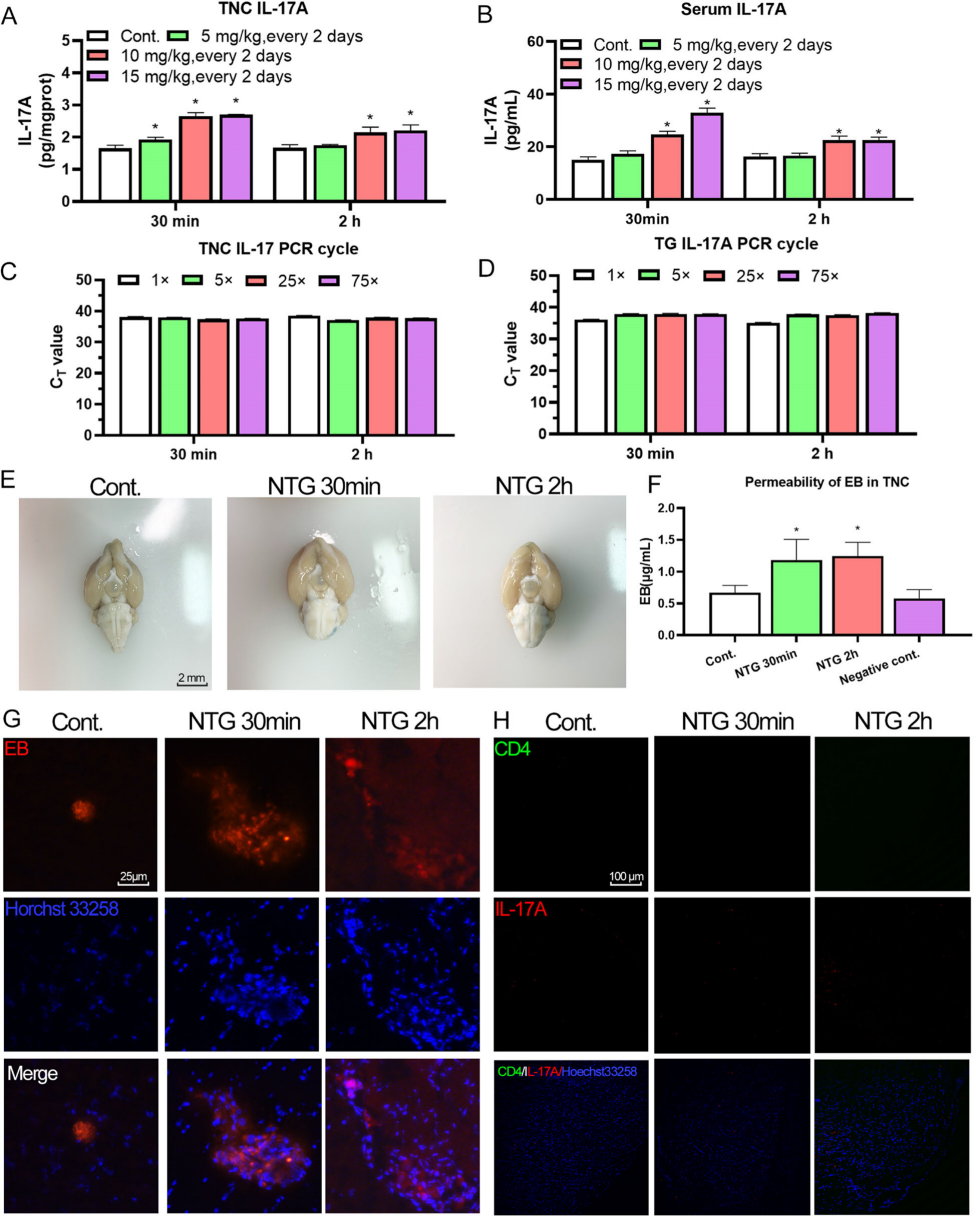
NTG-induced BBB alterations increased peripheral IL-17A access to the TNC. NTG administration increased IL-17A levels, while its transcription was not initiated in the TNC. (A and B) Content of IL-17A in the TNC and serum of rats with NTG-induced migraine. (C and D) The Ct value of the IL-17A mRNA in the TNC and TG of rats with NTG-induced migraine. NTG administration altered BBB permeability. (E) Bright-field image of EB permeation in rats with NTG-induced migraine. (F) EB permeability in the TNC of rats with NTG-induced migraine. (G) Images of EB staining in the TNC of rats with NTG-induced migraine. NTG administration did not induce CD_4_^+^/IL-17A^+^ T cell migration into the TNC. (H) Images of CD_4_ and IL-17A co-staining in rats with NTG-induced migraine. Statistical analysis was performed using one-way ANOVA, n = 3, *p < 0.05 compared to the control group

IL-17A has recently been at the forefront of neuroinflammation research. Emerging evidence indicates that IL-17A signals are the functional initiator of the inflammatory tendency of neurocytes [35, 51]. Interestingly, the IL-17A level was rapidly increased in the TNC and serum after NTG injection, whereas qRT–PCR data showed that IL-17A transcription was not initiated in TNC (Fig. 9A-D). In general, the region of TNC in MO possesses a relatively fragile BBB. This evidence confirmed the hypothesis that NTG triggers neuroinflammation in the TNC by increasing the permeability of the BBB around the TNC, which promotes IL-17A transfer. EB staining assays showed that NTG increased the permeability of EB in the peripheral circulation and its transport to the TNC (Fig. 9E and F). Fluograms also revealed EB exudation from blood vessels, while CD_4_^+^/IL-17A^+^ T cells, an immune cell express IL-17A by reactivation, was not observed in the TNC (Fig. 8G and H). IL-17A is involved in inducing the production of the cytokines IL-1β, IL-6 and TNF-α. Previous ELISA results showed that IL-1β, IL-6 and TNF-α levels were also upregulated along with IL-17A (Fig. 5A-C), verifying that IL-17A triggered inflammation in the TNC. Collectively, NTG mediates the increase in BBB permeability around the MO to transfer IL-17A through the induction of the lipid transport function, neuroinflammation, and TNC activation, which is a novel mechanism of NTG-induced migraine that possibly bypasses the trigeminovascular system.

## Discussion

In this study, we confirmed that NTG (10 mg/kg, s.c., every 2 d for a total of 5 times) was the optimal condition to provoke migraine that resulted in mechanical hyperalgesia and migraine-like behavior. Furthermore, NTG exerted a pharmacological effect on inducing neuroinflammation and enhancing TNC activation, in which IL-17A permeated the BBB from the periphery to potentially initiate the inflammatory polarization of microglia. These findings provide support for the role of neuroinflammation in migraine. Additionally, IL-17A is a promising target involved in migraine and rats with NTG-induced migraine might serve as a useful neuroinflammatory tool to investigate the interventions for migraine, including an anti-IL-17A strategy and protection of the BBB.

The Global Burden of Disease Study ranked migraine as the seventh most common disabling pathology among 369 diseases, referred to as the 7th disabler [3]. Tools for modeling the characteristics of sensory sensitivity in patients with migraine are indispensable to elucidate pathophysiological mechanisms and identify novel therapies for chronic migraine. A number of promising models have been developed, such as the nitroglycerin (NTG) model, inflammatory soup model, trigeminal allodynia model and FHM1R192Q transgenic model, for migraine studies [46]. Relative to episodic migraine, CM is the most burdensome form and is associated with significantly greater disability, higher rates of comorbidity, and increased direct and indirect costs [34]. However, the progression of migraine from an episodic to a chronic disorder in an inflammatory soup model and a FHM1R192Q transgenic model has been particularly difficult to study. The observation of the behaviors in migraine attacks require a long period to collect. Operating difficulties and high costs also limited their application. Thus, chronic intermittent administration of NTG is a controllable and sustained method to model multiple acute migraine attacks.

When using the i.p. routes of administration, a very high dose of NTG is needed to induce behaviors comparable to that of human migraine, which may lead to intense visceral pain stimulation because of the inflammatory exudation induced by hemangiectasis [14]. As expected, the migraine behavior of rats was difficult to evaluate after multiple NTG i.p. injections since visceral pain (writhing and cramping reaction) masked the migraine behavior in our preliminary test. Therefore, s.c. injection is an alternative method of administration to establish the model of chronic migraine and avoid migraine nonaction due to visceral stimulation. On the other hand, although abundant reports have attested that a single dose of 5–15 mg/kg NTG in rodents has been extensively applied to induce adventitious migraine pain after a single treatment, systemic research on the features of sensation and behavior in rats resulting from repeated administration of these doses of NTG at the same intervals is lacking, which was convenient for researchers to choose a model that parallels clinical conditions and identify potential mechanisms of migraine. Thus, relatively wide range of NTG doses (2.5–15 mg/kg) [5, 39] has been administered daily or on an alternate schedule to SD rats for optimization based on the migraine reaction and behavior. According to previous reports, five injections of 5 mg/kg NTG were required to establish a rat model with migrainous allodynia [25]; a higher dose of NTG (10 mg/kg) led to hyperalgesia [24, 40]. In this study, threshold testing showed that all conditions induced dose-dependent mechanical hyperalgesia, consistent with the previous literature. Although the high-dose group and consecutive group showed a relatively stable and low basic threshold, the behaviors of head scratching and cage climbing in the high-dose group (15 mg/kg every 2 d and 7.5 mg/kg daily) were not as sensitive as those in the 10 mg/mL, 2 d group. Nongradual migraine behavior was also observed in the low-dose group. Furthermore, the baseline threshold in the interval administration groups showed a fluctuating decrease, which was possibly implicated in the obvious migraine behavior. An inappropriate delivery method and dose might explain why only threshold results were extensively observed in the majority of studies. Thus, 10 mg/kg NTG administered s.c. every 2 d is a relatively optimized condition for establishing a model of chronic migraine in SD rats.

Dural neurogenic inflammation has been accepted as an important driver of migraine attacks for decades. Classic neurogenic inflammation is triggered by three main components, including the release CGRP and SP from meningeal afferent fibers, vasodilatation with plasma protein extravasation and meningeal mast cell degranulation [7, 9, 26, 29]. Another focus of migraine research has been the possible role of neurogenic neuroinflammation in a series of vital brain regions involved in migraine, including the thalamus, MO and cortex [19], although some inflammatory responses were not observed in a clinical trial [18]. In previous studies, the activation of neurogenic inflammation was indicated to be a crucial step in NTG-induced migraine attacks. Although the release of NO from NTG to activate its high-affinity receptor and trigger a cascade effect of cGMP leading to CGRP release is a well-accepted process by which NTG initiates neurogenic inflammation [6, 16], little evidence is available showing that meningeal neurogenic inflammation is the origin of genuine migraine attacks [18]. Recent fMRI data show that a migraine attack most likely originates in the hypothalamus and results in the activation of the MO [42]. In our rat model, the increases in CGRP and c-Fos levels revealed TNC activation. The NF-κB signaling cascade is involved in the upregulation of the migraine-related protein CGRP [54]. Thus, the increased levels of TNF-α, IL-6 and IL-1β, the activation of NF-κB signaling and polarized microglia indicated that neurogenic neuroinflammation contributes to TNC activation, suggesting that NTG might trigger an upstream mechanism in which neuroinflammation is involved in mediating migraine attack. Our further observations that NTG-induced neuroinflammation are blocked by ibuprofen is supported by similar outcomes in which NTG-induced hyperalgesia is abolished by the administration of nimesulide (a COX-2 inhibitor) both in animal and human studies [50]. This evidence suggests that neurogenic neuroinflammation characterized by increased CGRP and c-Fos levels is a compelling feature of the NTG model, which identified the fields of application of this model to test novel antiphlogistic or anti-CGRP therapies in individuals with migraine.

IL-17A is one of the most frequently investigated cytokines in IL-17 family, and it mediates the communication between immune cells and tissue. According to recent investigations, IL-17A plays a pivotal role in neuroinflammation [51]. Our observations showed that NTG increased IL-17A levels both in periphery and MO, which indicated that IL-17A might initiate NTG-induced neuroinflammation. Little evidence is available that cells other than immune cells express IL-17A. As a rule, immune cells express IL-17A and IL-17F, and tissue cells, such as keratinocytes in the skin, epidermal cells in the gut or glial cells in the CNS, respond to it [37]. In addition to cells in the peripheral immune system, microglia was also a cell type that responds to IL-17A through the NF-κB pathway during neuroinflammation, such as multiple sclerosis [23]. The transcription of IL-17A in the TNC and TG was detected to investigate its source. Interestingly, the extremely high Ct value of IL-17A indicated a lack of IL-17A expression in the TNC and TG. In CNS autoimmunity, CD_4_^+^ T cells enter the subarachnoid space by crossing the blood–cerebrospinal fluid barrier and are reactivated by macrophages and dendritic cells into IL17^+^ T cells [23, 31]. We thus speculated that reactivated CD_4_^+^ T cells and peripheral IL-17A permeation are the potential sources of IL-17A in the CNS. Thus, we further monitored the BBB permeability by injecting an impermeable dye and measured the levels of BBB-related proteins. The results proved that NTG increased BBB permeability in the MO, while CD_4_^+^/IL17^+^ T cells were not observed in the MO. The MO is a region with a relatively weak BBB and enhances biochemical delivery [2]. Therefore, these findings confirmed the mechanism that the integrity of the BBB was altered by NTG or CGRP; subsequently, IL-17A derived from periphery induced neuroinflammation and hypersensitization in the TNC, which gain a deep insight to the IL-17A inflammatory signaling in NTG-induced migraine and revealed that anti-inflammation or BBB protection strategy warrant further research to investigate the availability in clinical treatment.

## Conclusions

**Fig. 10:**
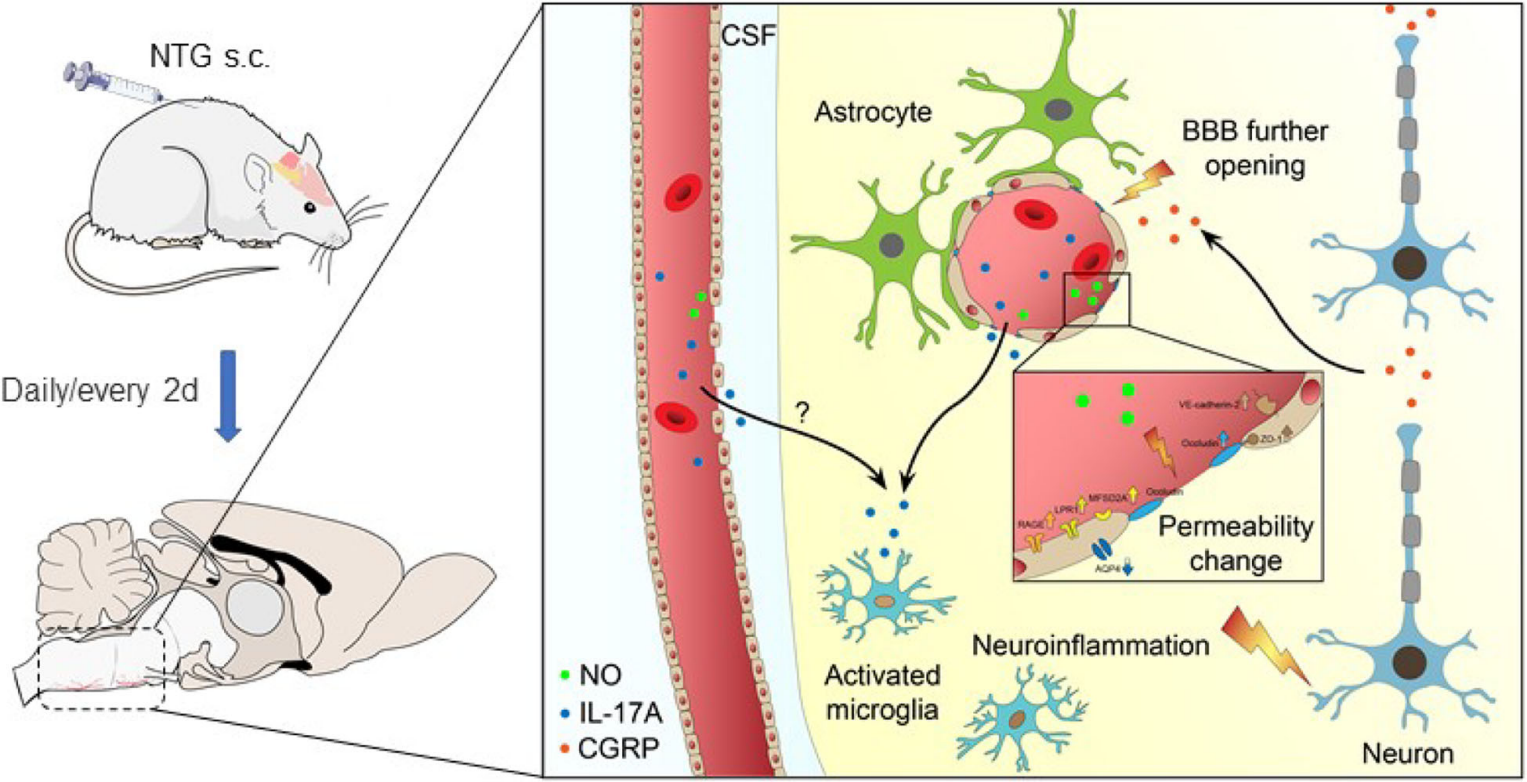
Peripheral IL-17A crosses BBB into MO with TNC to trigger neuroinflammation in rats with NTG-induced migraine

In summary, this study optimized and screened the conditions for establishing a chronic migraine model in rats. NTG (10 mg/kg, s.c., every 2 d for a total of 5 injections) was confirmed to provoke migraine resulting in mechanical hyperalgesia and migraine-like behavior. Moreover, neuroinflammation is a characteristic of the NTG-treated model, which can be alleviated by ibuprofen, suggesting that this model is capable of serving as a screening tool for migraine agents with potential anti-inflammatory activity. In terms of pathogenic mechanisms, NTG triggers CGRP-associated neuroinflammation by increasing BBB permeability and inducing the inflammatory polarization of microglia owing to IL-17A infiltration into the MO. These changes eventually lead to mechanical hyperalgesia and migrainous attack through TNC activation (Fig. 10).

## Data Availability

The datasets used and analyzed in the present study are available from the corresponding author on reasonable request.

## Abbreviations

NTG: Nitroglycerin
TNC: Nucleus caudate of trigeminal nerve
TG: Trigeminal ganglion
MO: Medulla oblongata
CGRP: Essential oil from *Eupatorium odoratum* L
NF-κB: Nuclear factor kappa-light-chain-enhancer of activated B cells
IL-17A: Interleukin-17A
i.p: Intraperitoneal injection
s.c: Subcutaneous injection
BBB: Blood-brain barrier
